# Maintenance of transposon-free regions throughout vertebrate evolution

**DOI:** 10.1186/1471-2164-8-470

**Published:** 2007-12-20

**Authors:** Cas Simons, Igor V Makunin, Michael Pheasant, John S Mattick

**Affiliations:** 1 Australian Research Council Special Research Center for Functional and Applied Genomics, Institute for Molecular Bioscience, University of Queensland, St Lucia QLD 4072, Australia

## Abstract

**Background:**

We recently reported the existence of large numbers of regions up to 80 kb long that lack transposon insertions in the human, mouse and opossum genomes. These regions are significantly associated with loci involved in developmental and transcriptional regulation.

**Results:**

Here we report that transposon-free regions (TFRs) are prominent genomic features of amphibian and fish lineages, and that many have been maintained throughout vertebrate evolution, although most transposon-derived sequences have entered these lineages after their divergence. The zebrafish genome contains 470 TFRs over 10 kb and a further 3,951 TFRs over 5 kb, which is comparable to the number identified in mammals. Two thirds of zebrafish TFRs over 10 kb are orthologous to TFRs in at least one mammal, and many have orthologous TFRs in all three mammalian genomes as well as in the genome of *Xenopus tropicalis*. This indicates that the mechanism responsible for the maintenance of TFRs has been active at these loci for over 450 million years. However, the majority of TFR bases cannot be aligned between distantly related species, demonstrating that TFRs are not the by-product of strong primary sequence conservation. Syntenically conserved TFRs are also more enriched for regulatory genes compared to lineage-specific TFRs.

**Conclusion:**

We suggest that TFRs contain extended regulatory sequences that contribute to the precise expression of genes central to early vertebrate development, and can be used as predictors of important regulatory regions.

## Background

Vertebrate genomes are typically densely packed with transposable elements that can account for almost half of all genomic DNA. Historically thought of as only parasitic elements, recently it has become clear that the influence of transposons on genomic structure and function in vertebrates is complex [1-4]. For instance it has been shown that many transposon-derived sequences have been exapted into functional roles such as regulatory elements controlling gene expression [5] and contributing to proteome diversity through introduction of cryptic splice sites and exonization [6]. Alternatively, transposon insertion into functional DNA can be deleterious as is demonstrated by the event of genetic diseases resulting from *de novo *transposon insertions [7]. Nevertheless, transposons are pervasive features of mammalian genomes (*e.g*., the human genome contains over three million transposons with an average spacing of 472 bp between them), suggesting that the majority of insertion events are easily tolerated and are not significantly deleterious to the host. We recently described the presence of regions between 10 and 80 kb long in the human, mouse and opossum genomes that appear to be unable to tolerate any transposon insertions which suggests these regions are either densely packed with discrete functional elements or represent unusually long functional units [8].

These transposon-free regions (TFRs) cannot be explained using random models of transposon insertion, and are often associated with genes that play important roles in development, such as members of the *HOX, SOX, FOX *and *TBX *gene families [8,9]. Although most human, mouse and opossum transposon insertions have occurred independently in each linage, the majority of large human TFRs are also transposon-free in the orthologous regions of the mouse and opossum genomes [8]. TFRs have been maintained at many loci at least since the last common ancestor of placental mammals and marsupials, about 185 million years [10]. Intriguingly, all members of several gene families, such as the *HOX *clusters and the *IRX *family, are contained within TFRs. This suggests that the mechanism responsible for the maintenance of TFRs may have been active for over 500 million years, since the gene duplication events that originally gave rise to these families [11]. To investigate this possibility we looked for TFRs in more distant vertebrate lineages whose genomes have since been sequenced, i.e., fish and frog.

Here we report 470 transposon-free regions ≥ 10 kb and 4,891 TFRs ≥ 5 kb in zebrafish demonstrating that TFRs are a common feature of vertebrate genomes. Many zebrafish TFRs are orthologous to TFRs in mammals and frog suggesting these TFRs have been present and maintained since the dawn of the vertebrate lineage.

## Results

Of the three sequenced fish genomes, only the zebrafish genome exhibits a high density of transposons. The zebrafish genome is ~1.6 Gb in size and contains over 1.4 million annotated transposons, with an average spacing of approximately 800 bp (see Methods). We identified the longest segments of the zebrafish genome that lacked any recognizable transposons. We also repeated and updated our TFR analysis on the latest releases of the human, mouse and opossum genomes. All four genomes were analyzed for TFRs using the methodology described previously [8]. Briefly, all regions larger than 5 kb free of any annotated transposons or genome assembly gaps were identified. We then excluded any regions that contained > 20% non-transposon repeat sequence, homology to the mitochondrial genome, or showed evidence of having undergone recent expansion (e.g. tandem repeats of complex DNA).

Examination of the zebrafish genome revealed a small number of loci containing TFRs that appear to be duplicated and often separated by a gap in the genome assembly. Although many of these may represent true genomic duplications it is also possible that some may result from artifacts in the current draft genome assembly. We eliminated all potential duplicates by making pairwise alignments of all zebrafish TFRs and excluding one member of each pair from all further analysis (see Methods).

We identified a final dataset of 470 zebrafish TFRs ≥ 10 kb and 4,891 TFRs ≥ 5 kb, intermediate between the 396 opossum and 856 human TFRs ≥ 10 kb (Table 1 and Additional Files 1, 2, 3, 4). Using a simple model of random transposon insertion, we calculated that the zebrafish genome would be expected to contain only six TFRs ≥ 10 kb and 2,778 TFRs ≥ 5 kb (see Methods). We estimate the probability of the observed 470 TFRs occurring by chance of is *P *< 10^-285 ^and *P *< 10^-300 ^for observing 4,891 TFR ≥ 5 kb.

**Table 1:** Counts of TFRs in four vertebrate genomes

	≥ 10 kb		≥ 5 kb	
		
	Number	Size (bp)	Number	Size (bp)
Zebrafish	470	6,202,556	4,891	34,677,352
Human	856	12,090,440	9,203	65,097,113
Mouse	1,112	15,136,372	14,154	97,707,658
Opossum	396	5,334,925	4,818	33,312,949

All but one of the 15 longest zebrafish TFRs overlap a gene, ten of which encode transcription factors (Table 2), and the human homologs of each of these transcription factor genes are also associated with TFRs ≥ 10 kb. In addition to the transcription factor gene-associated TFRs, the remaining TFRs are also associated with a number of other regulatory genes, including those encoding the hedgehog receptor Ptc1 [12] and the oncogenic histone methyltransferase Mll2 [13].

**Table 2:** The fifteen longest TFRs in zebrafish

TFR ID	Genomic position	Size (bp)	Overlapping genes^a^	TFR in human^b^
dr23.144	chr23:35,638,710-35,705,409	66,700	*hoxc5a-11a*	Yes
dr3.77	chr3:22,940,664-22,984,418	43,755	*hoxb2a-4a*	Yes
dr19.54	chr19:13,924,211-13,955,334	31,124	*hoxa1a-5a*	Yes
dr4.159	chr4:16,685,114-16,714,306	29,193	*plxna4*	No
dr5.225	chr5:57,548,679-57,577,252	28,574	*nr2f1*	Yes
dr15.69	chr15:25,034,169-25,061,004	26,836	*-*	-
dr3.89	chr3:24,406,349-24,432,207	25,859	IGF2BP3	Yes
dr3.78	chr3:22,984,979-23,010,566	25,588	*hoxb5a-b8a*	Yes
dr4.190	chr4:18,669,203-18,694,678	25,476	*sox5*	Yes
dr6.213	chr6:59,099,468-59,123,108	23,641	*barhl2*	Yes
dr2.134	chr2:31,040,372-31,063,335	22,964	*ptc1*	Yes
dr24.53	chr24:17,570,229-17,592,632	22,404	*bmi1, commd3*	Yes
dr23.116	chr23:31,417,962-31,439,642	21,681	MLL2	No
dr9.78	chr9:21,302,333-21,323,748	21,416	ZFHX1B	Yes
dr15.179	chr15:41,033,477-41,054,729	21,253	*tbx2b*	Yes

^a ^Zebrafish genes are listed in lower case italic; human proteins mapped to corresponding loci in the zebrafish genome are given in upper case.

^b ^The orthologous gene in human overlaps a TFR ≥ 10 kb.

Similar to that previously observed in mammals [8], zebrafish TFRs are strongly associated with genes, 78% of TFRs ≥ 10 kb (62% of TFRs ≥ 5 kb) overlap a gene, although only 14% (11%) of TFR bases are annotated as exonic. Furthermore, when a TFR does overlap a gene, the TFR typically extends 5' of the transcription start site into the upstream intergenic region (76% of genic TFRs ≥ 10 kb and 58% ≥ 5 kb).

### The majority of zebrafish TFRs have orthologous TFRs in mammals

We mapped zebrafish TFRs to syntenic regions of the human genome to examine whether the high level of orthology observed between the longest fish and human TFRs extended to smaller TFRs. Each zebrafish TFR was mapped to a single locus in the human genome using the UCSC whole genome alignments to identify the best alignment covering the greatest number of bases in each TFR (see Methods). The individual blocks within the selected alignment were then mapped to the human genome. We considered TFRs orthologous between zebrafish and human, mouse, or opossum, if any zebrafish TFR contained blocks of alignment within a human, mouse or opossum TFR.

**Figure 1:**
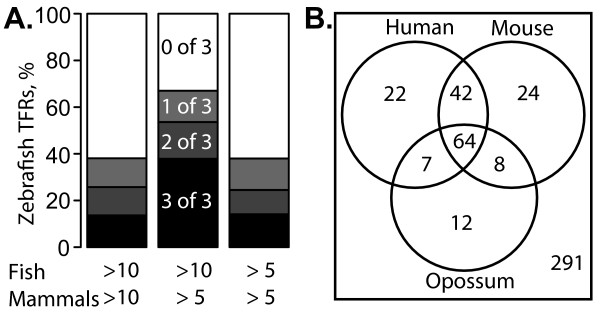
**Summary of the number of zebrafish TFRs that are orthologous to TFRs in three mammalian species**. (A) Proportions of zebrafish TFRs that have an orthologous TFR in three, two or one mammal species. The bar on the left relates to zebrafish TFRs ≥ 10 kb with orthologous mammalian TFRs ≥ 10 kb. The center bar relates to zebrafish TFRs ≥ 10 kb with orthologous mammalian TFRs ≥ 5 kb. The bar on the right relates to zebrafish TFRs ≥ 5 kb with orthologous mammalian TFRs ≥ 5 kb. (B) Venn diagram of zebrafish TFRs ≥ 10 kb with orthologs ≥ 10 kb in one or more mammal species. The numbers on the graph represent the count of zebrafish TFRs in each category.

We were able to identify orthologous human TFRs for 54% of zebrafish TFRs ≥ 10 kb. The majority of these (135 of 253) have orthologs ≥ 10 kb in human. Similar numbers of orthologous TFRs were found in each of the mammalian species such that 315 (67%) of TFRs ≥ 10 kb have an ortholog ≥ 5 kb and 179 (38%) have an ortholog ≥ 10 kb in one or more mammals (Fig. 1). Furthermore, 1270 (26%) zebrafish TFRs ≥ 5 kb have an ortholog in human and 1858 (38%) have an ortholog in one or more mammals. The synteny between such a large number of fish and mammal TFRs is a clear indication that in many of these loci, TFRs have been maintained throughout vertebrate evolution.

**Figure 2:**
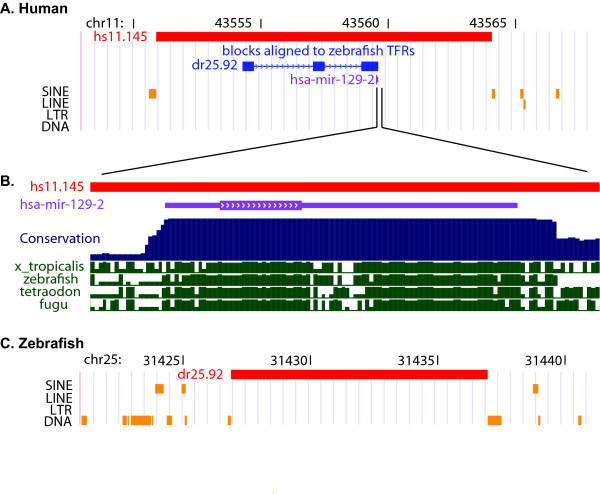
**Orthologous human and zebrafish TFRs that contain the miRNA mir-129-2**. (A) 20 kb of the human genome (chr11:43,548,001–43,568,000) including the non-genic 13 kb TFR hs11.145 (red bar). Thick blue bars indicate blocks of sequence that are alignable to the orthologous zebrafish TFR dr25.92. Small purple bar indicates the position of the human miRNA mir-129-2. (B) A close up view of 130 bp around mir-129-2, thick purple bar indicates the mature miRNA, thin purple line indicates pre-miRNA hairpin. Blue conservation plot is based on the alignment of 17 vertebrate species and green plot based on pairwise alignment of human and zebrafish that shows a conservation profile consistent with the presence of a miRNA conserved in each species [38]. (C) Syntenic region of the zebrafish genome (20 kb chr25:31,421,001–31,441,000) including the TFR dr25.92. Thick blue bars indicate blocks of sequence that are alignable to the orthologous human TFR hs11.145. Although there are currently no genes annotated in this region, the conservation profile suggests that an ortholog of mir-129-2 resides within the TFR. All images are modified screen shots taken from the UCSC genome browser [31].

Small blocks of alignment that cover exons of protein coding genes anchor the majority of orthologous TFRs pairs. However, 16% of orthologous zebrafish TFRs ≥ 10 kb do not contain exons of known genes in zebrafish nor align to exons in human. For example, the 10 kb TFR dr25.92 and its human ortholog hs11.145 lack any protein coding genes but the latter centers over the microRNA locus *mir-129-2*. (Fig. 2). In another example, the 9.6 kb and 11 kb zebrafish TFRs dr14.213 (chr14:65,616,526-65,626,230) and dr14.214 (chr14:6,564,1118-65,652,778) are in the center of a 158 kb intergenic region, and the orthologous human TFRs, hsX.261 (chrX:136,142,873-136,149,206) and hsX.262 (chrX:136,178,986-136,185,924), are found in the center of a 500 kb gene desert. Interestingly the border of both the human and zebrafish intergenic regions is adjacent to the zinc finger transcription factor gene *ZIC3*.

While only 5.2% of the non-transposon-derived portion of the zebrafish genome can be aligned to the human genome, 24% of bases within zebrafish TFRs with orthologous human TFRs ≥ 10 kb are alignable (19% of TFR ≥ 5 kb bases), representing a 4.6 fold enrichment. However, the remaining three quarters of TFR bases cannot be aligned to mammalian genomes and, thus it seems clear that conservation of primary sequence alone cannot explain the selection pressure against transposon insertion in these regions.

There is also evidence of lineage-specific TFRs. For example, the 17 kb zebrafish TFR dr5.202 almost entirely spans the ZFR gene, whereas there are no TFRs ≥ 5 kb within or near the ZFR orthologs in the human or mouse genomes. One quarter of zebrafish TFRs ≥ 10 kb have no orthologous TFR in mammals.

### No correlation in GC content between orthologous TFRs

**Figure 3:**
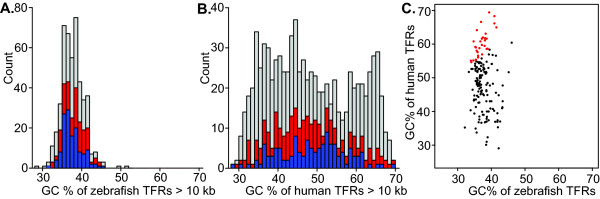
**Comparison of the GC content of TFRs in zebrafish and human**. (A) Histogram of the GC content of zebrafish TFRs. Area indicated in blue describes the subset of TFRs that have an orthologous TFR in human larger than 10 kb, the area in red have an orthologous TFR in human larger than 5 kb. (B) Histogram of the GC content of human TFRs. Area indicated in blue describes the subset of TFRs that have an orthologous TFR in zebrafish larger than 10 kb, the area in red have an orthologous TFR in zebrafish larger than 5 kb. (C) Scatter plot of the GC content of orthologous pairs of zebrafish and human TFRs ≥ 10 kb in both species. Points in red indicate TFR pairs with a difference of absolute GC% greater than 20.

In light of our previous observation that certain characteristics of mammalian TFRs may be related to the GC content of the TFR [8], we compared the GC content of zebrafish and human TFRs. Zebrafish TFRs have a GC content distribution ranging from 29% to 52% (average 38%), in contrast to the very broad GC content distribution of human TFRs which ranges from 29% to 69% (Fig. 3). There is no apparent correlation between the GC content of pairs of orthologous human and zebrafish TFRs (Fig. 3C), although human TFRs tend to have a higher GC content than their zebrafish ortholog. This is most dramatic in the 17% of orthologous pairs of TFRs where the absolute difference in GC percent is greater than 20 (Fig. 3C). For example, the orthologous TFRs dr5.101 (chr5:30,517,101-30,528,546) and hs22.13 (chr22:18,119,370-18,133,892), both of which are greater than 11 kb long and extend over the 5' half of the T-box transcription factor gene *TBX1 *(see Additional file 6, Fig. S1), have GC contents of 35% and 64%, respectively. The presence of such large differences in GC content and the lack of any correlation between GC content in orthologous pairs provides further evidence that TFR primary sequence may be under different selection pressures and/or rapid drift while maintaining their refraction to transposon insertions.

### A core set of ancient TFRs is common to all vertebrate lineages

**Figure 4:**
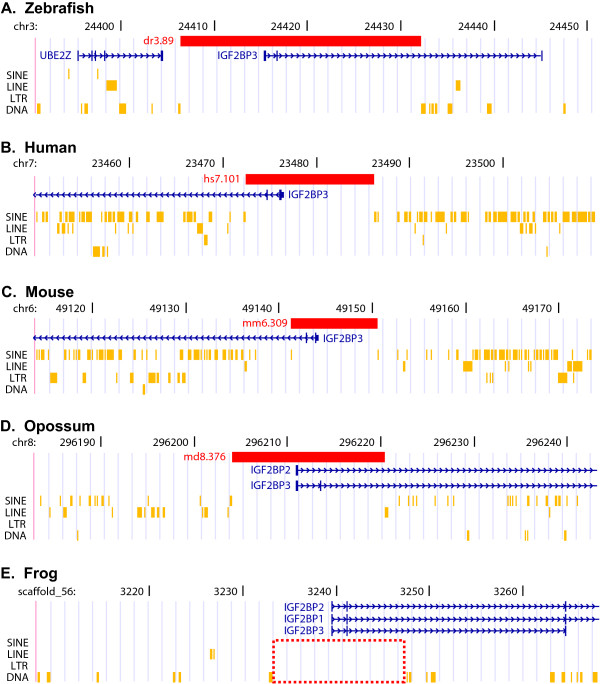
**Zebrafish transposon-free region dr3.89 and the orthologous regions of four vertebrate species**. Each panel shows a modified screenshot displaying a 60 kb region from the UCSC genome browser. Horizontal red bars indicate TFRs and brown ticks indicate transposons. (A) Zebrafish (chr3:24,391-24,451 kb, March 2006) including the 25.9 kb TFR dr3.89. Human proteins mapped to the zebrafish genome by chained tBLASTn are indicated in blue. (B) Human (chr7:23,450-23,510 kb, March 2006) including the 13.7 kb TFR hs7.101. Human RefSeq genes are indicated in blue. (C) Mouse (chr6:49,114-49,174 kb, February 2006) including the 9.3 kb TFR mm6.309. Mouse RefSeq genes are indicated in blue. (D) Opossum (chr8:296,183-296,243 kb, January 2006) including the 16.4 kb TFR md8.376. Human RefSeq genes mapped to the opossum genome with BLAT are indicated in blue. (E) Frog (scaffold_56:3,208-3,268 kb, August 2005) including a 14 kb region that contains no transposons (red box). Human proteins mapped to the frog genome with tBLASTn are indicated in blue.

Our analysis of the human, mouse, opossum and zebrafish genomes identified a core group of TFRs that are common to these four divergent vertebrate species. For example, the regions orthologous to the 25 kb zebrafish TFR dr3.89, which overlaps the *insulin-like growth factor II mRNA-binding protein 3 (igf2bp3) *gene, are also transposon-free in human, mouse, opossum and frog (Fig. 4). Sixty-four (14%) zebrafish TFRs have orthologs ≥ 10 kb in all three mammal species, and 178 (38%) have orthologous TFRs ≥ 5 kb. We identified a further 690 (14%) zebrafish TFRs ≥ 5 kb that also appear to have been maintained transposon-free since the last common ancestor of ray-finned fish and mammals.

Although the current assembly of the frog (*Xenopus tropicalis*) genome is not of sufficient quality to allow a full genome analysis of TFRs, we identified the orthologous regions of each of the 64 zebrafish TFRs ≥ 10 kb that were also present in mouse, opossum, and human. In 58 of 64 cases we were able to identify orthologous regions of greater than 10 kb that contained no annotated transposons, although 10 contain one or more assembly gaps (see Additional file 5, Table S1). Four of the remaining 6 TFRs map to transposon-free regions of at least 8.7 kb. This suggests that the majority of these ancient TFRs have been maintained transposon-free throughout the evolution of all major vertebrate lineages.

### Ancient TFRs are enriched for regulatory genes compared to lineage-specific TFRs

Table 2 suggests that transcription factor genes are more likely to be associated with syntenically conserved TFRs in zebrafish and human than other categories of genes. To generalize this observation we looked for Gene Ontology (GO) categories and InterPro domains that are significantly enriched in genes associated with these ancient TFRs that are common to both zebrafish and mammals. To take advantage of the depth of gene annotations available in human, we analyzed the GO associations for all human TFRs with orthologous TFRs in zebrafish, mouse and opossum.

Of the 572 human TFRs ≥ 5 kb with orthologous TFRs in all species, 42% overlap a total of 234 genes annotated as "regulation of transcription", representing a 3.6 fold enrichment (*P *< 10^-87^). Seventy percent of TFRs ≥ 10 kb, overlap a total of 62 "regulation of transcription" genes, representing a 5.4 fold enrichment (*P *< 10^-41^). Although genes annotated as "regulation of transcription" are over represented generally in all human TFRs ≥ 10 kb, the level of enrichment (2.7 fold, *P *< 10^-58^) is half that observed for TFRs that have orthologs in all species. This strongly suggests that TFRs retained in multiple lineages are more likely to be associated with regulatory genes.

Higher enrichment can be seen in the related but more specific annotation "transcription factor activity" that shows up to a 12.8 fold enrichment *(P *< 10^-57^) in TFRs ≥ 10 kb with orthologs in all species. Genes with an InterPro annotated homeobox domain were also highly enriched in these ancient TFRs. A total of 85 of ancient TFRs ≥ 5 kb overlap 94 genes containing a homeobox domain, yielding a 14 fold enrichment (*P *< 10^-80^), and represents 22% of all homeobox genes in the human genome.

## Discussion

Here we have described a large number of regions within the zebrafish genome that lack any annotated transposons, demonstrating that transposon-free regions are not restricted to mammals but are a common feature of vertebrate genomes.

Furthermore, we have shown that many TFRs have been present since the dawn of the vertebrate lineage, and that these TFRs are significantly associated with developmental genes such as those encoding homeobox-containing proteins and transcription factors.

A potential problem when examining TFRs within any one lineage is that it can be difficult to distinguish short regions that have been selectively maintained transposon-free, as opposed to those that have a low transposon density by chance. However, we identified 690 zebrafish TFRs ≥ 5 kb that have orthologous TFRs in all three mammalian species examined and 1,858 that have an ortholog in at least one mammal. This provides strong evidence that a significant subset of shorter TFRs are also under functional constraint. Furthermore, the increased enrichment of syntenically conserved TFRs associated with regulatory genes compared to lineage-specific TFRs suggests that it may be useful to separate these two classes of TFRs when assessing the potential functional importance of a TFR.

The vast majority (74%) of zebrafish insertions are DNA transposons (class II transposons), compared to the retrotransposons (class I transposons) that dominate the mammal genomes (94% of human insertions). As the majority of TFRs have been maintained at the same loci in both species through independent infestation of their genomes with these different classes of transposons we can conclude that TFRs are resistant to transposons of both major classes of transposable elements. In our previous analysis of mammalian TFRs we speculated that there are two general mechanisms by which these regions may be maintained: the underlying sequence may be resistant to transposon insertion, or transposon insertion in these regions may be deleterious and therefore subject to strong negative selection. As DNA transposons and retrotransposons have substantially different mechanisms of transposition [14], and given that much of the primary sequence of these regions has undergone significant change, it would seem unlikely that a molecular inhibition is in place that is capable of restricting insertion by such a wide range of mechanisms over such extended regions of sequence. This alternative is supported by the observation that in cancer-associated retroviral screens the integration of retroviruses, which occurs by a mechanism somewhat similar to retrotransposon integration [15], appears to be uninhibited within TFRs [8]. Given these results, we suggest that the existence of TFRs is more likely to be the result of strong evolutionary selection against the interruption of these regions by transposon-derived sequences, rather than a mechanism that precludes such insertions from occurring in the first place.

Although TFRs are enriched for conserved DNA, 75% of bases within zebrafish TFRs cannot be aligned, let alone show homology, to mammalian genomes, suggesting that primary sequence conservation alone cannot account for the presence of TFRs. Moreover the dramatic changes in GC content (up to 1.8 fold increase) in orthologous pairs of TFRs suggest that some orthologous pairs have undergone different evolutionary histories that have reshaped the primary sequence composition of these regions either because of different selection pressures associated with phenotypic radiation and/or because of rapid drift. Our analysis is consistent with the recent reports that regulatory function can be conserved between species without the requirement of primary sequence similarity [16,17].

The maintenance of many TFRs throughout vertebrate evolution and their strong association with many key regulators of early development are a clear indication of the importance of TFRs in the genome. However, the molecular and genetic basis that prevents these extended regions from tolerating transposon sequence is still unclear. A recent analysis of chromatin domains in the mouse genome may suggest a biological explanation for at least some TFRs. Bernstein et al (2006) analyzed chromatin patterns in mouse embryonic stem (ES) cells across 61 regions covering ~2.5% of the genome. They compared the distribution of histone H3 Lys4 (Lys4) methylation, typically associated with transcriptonally active chromatin, and histone H3 Lys27 (Lys27) methylation, typically associated with transcriptionally silent chromatin. A strong association was observed between Lys27 chromatin domains and "transposon exclusion zones", which were defined using criteria similar to TFRs. In the regions analyzed, 95 of the 143 (66%) Lys27 chromatin domains identified contain at least one TFR ≥ 5 kb, many of which also contained Lys4 chromatin domains. These novel chromatin structures, occupied concurrently by both "repressive" Lys27 and "activating" Lys4 chromatin modifications were termed "bivalent domains" and are believed to maintain many developmentally important genes in a transcriptionally repressed state in ES cells but poised for immediate activation when the correct developmental cues are received [18].

As transposon-derived sequences are known to attract repressive epigenetic modifications [19-21] it is possible to envisage a mechanism whereby insertion of transposon sequence in these chromatin domains attracts additional modifications and prevents the regulatory domain from functioning correctly. However, the association between transposon free regions and the chromatin domains examined was not observed in cells that have undergone differentiation [16,18], suggesting an alternative model where the absence of repeats is itself an important marker for epigenetic reprogramming and establishes these complex chromatin domains during germ cell development and early embryogenesis [22].

It must be noted that some bivalent domains exist in regions that contain substantial amounts of transposon sequence (up to 23% of bases), and reciprocally, many TFRs do not coincide with any of the chromatin domains identified by this analysis. This demonstrates that our understanding of the relationship between these chromatin structures and TFRs is far from complete, and that the reasons for their occurrence are not entirely congruent. It would be interesting to directly compare the chromatin status across homologous zebrafish and mouse TFRs, particularly in those regions with high levels of primary sequence divergence.

## Conclusion

We suggest that TFRs are distinct genomic signatures that may be useful for rapid prediction of important regulatory modules in vertebrate genomes. Furthermore, our results suggest that the analysis of non-random patterns of different classes of sequences within genomes, in contrast to the traditional focus on primary sequence conservation, may offer new opportunities for the detection of functional elements within the genome.

## Methods

### Transposon annotations

Genomic coordinates of all regions annotated as transposon derived sequence were downloaded for each genome from the UCSC genome browser [23]. The annotations were generated by the UCSC genome browser team using the RepeatMasker program [24] that scans genomic sequence for regions of significant homology to the RepBase library of repeat sequences [25] and the output can be visualized within the browser as the "RepeatMasker" track. For the purposes of this analysis, all annotations from the classes DNA, LINE, SINE, and LTR were considered transposons.

### Identification of TFRs

TFRs were identified and filtered as previously described [8]. The zebrafish data sequence data were produced by the *Danio rerio *(zebrafish) Sequencing Group at the Sanger Institute [26]. Genome assemblies used were: zebrafish – danRer4 (Zv6) [27], human – hg18 (NCBI build 36.1) [28], mouse – mm8 (NCBI build 36) [29] and opossum – monDom4 [30].

To remove any TFRs in the zebrafish dataset that may be the result of artifactual assembly duplications [27], all zebrafish TFRs were aligned against each other using BLAT [31] using default parameters with a minScore = 2000 and a self generated ooc file. All cases were identified where greater that 90% of a TFRs bases were identical to another TFR. For each pair of potentially duplicated TFRs, the shortest of the pair was removed from further analysis (see Additional file 5, Table S2).

### Estimation of expected number of TFRs in the genome

To estimate the expected number (μ) of TFRs in the zebrafish genome we used the following formula:

μ=n×e−ndN
 MathType@MTEF@5@5@+=feaagaart1ev2aaatCvAUfKttLearuWrP9MDH5MBPbIqV92AaeXatLxBI9gBaebbnrfifHhDYfgasaacPC6xNi=xI8qiVKYPFjYdHaVhbbf9v8qqaqFr0xc9vqFj0dXdbba91qpepeI8k8fiI+fsY=rqGqVepae9pg0db9vqaiVgFr0xfr=xfr=xc9adbaqaaeGacaGaaiaabeqaaeqabiWaaaGcbaacciGae8hVd0Maeyypa0JaemOBa4Maey41aqRaemyzau2aaWbaaSqabKqbagaadaWcaaqaaiabgkHiTiabd6gaUjabdsgaKbqaaiabd6eaobaaaaaaaa@3946@

Where *N *= the total number of bases between transposons, n = the number of transposons, and *d *= the minimum size of TFR (eg 10,000 bp). The probability of finding the observed number of TFRs was estimated using the Poisson distribution with parameter *μ*. To overcome the problem that many transposons are found nested within previous insertions, uninterrupted blocks of transposon-derived sequence consisting of one or more separate transposon annotations, were considered a single unit when counting the total number of transposons in the genome.

### Identification of orthologous TFRs between species

To identify the orthologs of zebrafish TFRs we used the whole genome alignment nets provided by the UCSC genome browser [32]. For each TFR we identified the single alignment net that contained the greatest number of alignable TFR bases, this ensured that each TFR could be mapped to no more than one loci in the second genome. Each block of TFR bases that were aligned in the selected net alignment was then mapped to the second species using the UCSC genome browser "liftOver" utility using default parameters [31]. If any of the mapped alignment blocks overlapped with a TFR in the second species this pair of TFRs were considered orthologous.

### Gene annotations

Ensembl (v42) Gene Predictions [33] were used for analysis of the zebrafish genome and UCSC "Known Gene" annotations were used for the human genome. Both sets of gene annotations were obtained from the UCSC genome browser [34,31].

### Gene Ontology/InterPro enrichment and P-values

GO annotations were taken from the September 2006 EMBL GOA Uniprot database [35] and the September 2006 GO schema [36]. Known Isoforms identifiers for UCSC Known Genes were used to make sure one gene was only counted once where there were multiple isoforms. A Perl script and SQL code were created to calculate enrichment of terms and "Fisher's Exact" *P*-values against a background of all GO annotated genes in the UCSC Known Genes database. Any GO term with less than two-fold enrichment, or a *P*-value greater than 10^-15^, or less than 10 associated genes, was discarded. While we did not directly correct for multiple-hypothesis testing, in practice we performed less than 100 individual tests deeming the reported *P*-values highly significant.

InterPro annotations of all known genes were retrieved from the UCSC genome browser database. Enrichment of terms and "Fisher's Exact" *P*-values were calculated as for GO annotations above against a background of all InterPro annotated genes in the UCSC Known Genes database.

## Authors' contributions

CS carried out the bioinformatics analysis. CS and IVM conceived and designed the analysis and drafted the manuscript. MP wrote the gene ontology analysis program as well as several of the other tools used in the analysis, and he participated in the manuscript revision. JSM participated in the study design and coordination and participated in the manuscript preparation. All authors read and approved the manuscript.

## Supplementary Material

Additional File 1 **Chromosomal coordinates of zebrafish TFRs**. A tab delimited text file giving the danRer4/Zv6 chromosomal coordinates (Chromosome, start, end, ID) for all zebrafish TFRs. This data is also available in a browsable format with direct links to the UCSC genome browser at: Click here for file

Additional File 2 **Chromosomal coordinates of human TFRs**. A tab delimited text file giving the hg18/NCBI 36 chromosomal coordinates (Chromosome, start, end, ID) for all zebrafish TFRs. This data is also available in a browsable format with direct links to the UCSC genome browser at: Click here for file

Additional File 3 **Chromosomal coordinates of mouse TFRs**. A tab delimited text file giving the mm8/NCBI 36 chromosomal coordinates (Chromosome, start, end, ID) for all zebrafish TFRs. This data is also available in a browsable format with direct links to the UCSC genome browser at: Click here for file

Additional File 4 **Chromosomal coordinates of opossum TFRs**. A tab delimited text file giving the monDom4 chromosomal coordinates (Chromosome, start, end, ID) for all zebrafish TFRs. This data is also available in a browsable format with direct links to the UCSC genome browser at: Click here for file

Additional File 6 **Supplemental figure S1**. Orthologous pair of TFRs in zebrafish and human that have very different GC contents. (A) 30 kb region of the human genome chr22:18,108,001-18,138,000 enclosing the TFR hs22.13 (red bar, 63.9% GC) and the gene TBX1 shown in blue. (B) The syntenic region of zebrafish genome (chr5:30,507,001-30,537,000) showing the 11 kb TFR dr5.101 (35.4% GC) and the ortholog of human tbx1. Above each panel is a smoothed plot describing the percent GC content in 5 bp [31]. Both images are modified screen shots taken from the UCSC genome browser [31]. Click here for file

Additional File 5 **Supplemental tables S1 and S2**. Tables describing the presence of TFRs in *X. tropicalis *orthologous to the 58 zebrafish TFRs ≥ 10 kb found in all mammals and the pairs of potentially duplicated TFRs found in the zebrafish genome that were removed from the analysis. Click here for file
