# Supplementary figures and images for: Maintenance of transposon-free regions throughout vertebrate evolution
Source: BMC Genomics. 2007 Dec 20;8:470. doi: 10.1186/1471-2164-8-470 (PMC2241635; doi:10.1186/1471-2164-8-470)

## A. Human

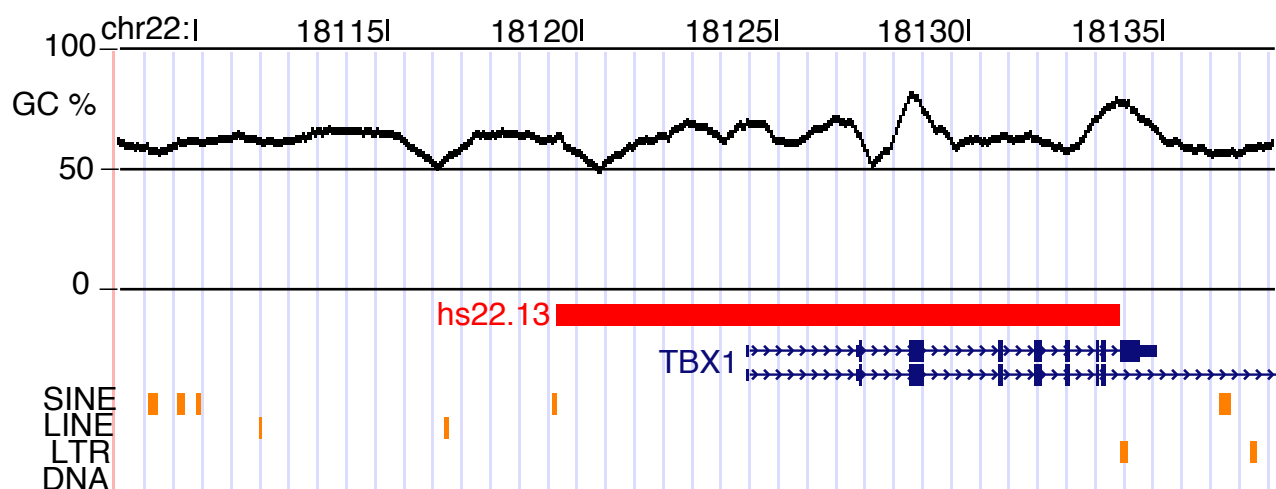

## B. Zebrafish

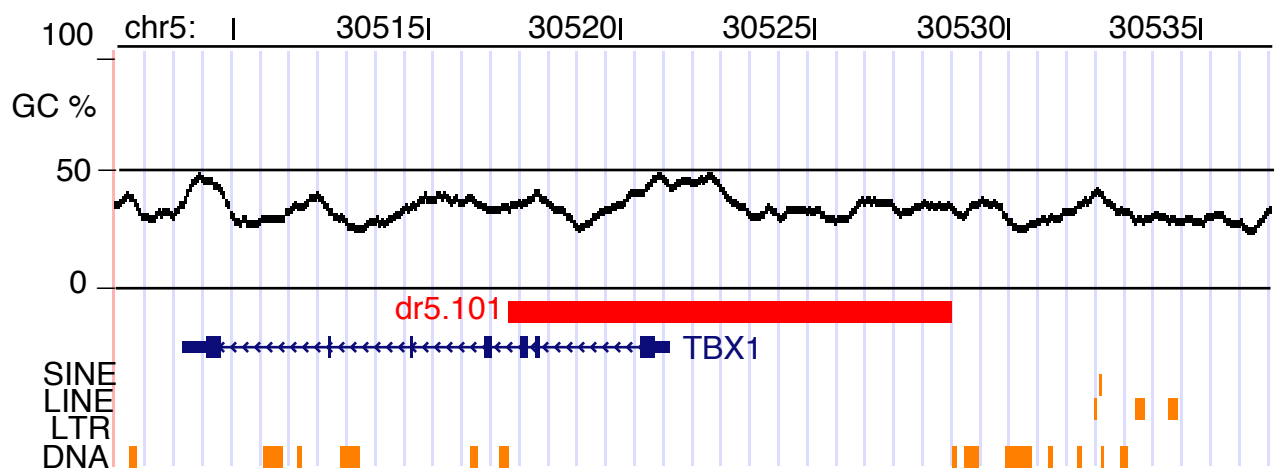

Supplement: Additional File 6 — Supplemental figure S1. Orthologous pair of TFRs in zebrafish and human that have very different GC contents. (A) 30 kb region of the human genome chr22:18,108,001-18,138,000 enclosing the TFR hs22.13 (red bar, 63.9% GC) and the gene TBX1 shown in blue. (B) The syntenic region of zebrafish genome (chr5:30,507,001-30,537,000) showing the 11 kb TFR dr5.101 (35.4% GC) and the ortholog of human tbx1. Above each panel is a smoothed plot describing the percent GC content in 5 bp [31]. Both images are modified screen shots taken from the UCSC genome browser [31]. [file 1471-2164-8-470-S6.pdf]
