# Supplementary material for: Maintenance of transposon-free regions throughout vertebrate evolution
Source: BMC Genomics. 2007 Dec 20;8:470. doi: 10.1186/1471-2164-8-470 (PMC2241635; doi:10.1186/1471-2164-8-470)
Supplement: Additional File 5 — Supplemental tables S1 and S2. Tables describing the presence of TFRs in X. tropicalis orthologous to the 58 zebrafish TFRs ≥ 10 kb found in all mammals and the pairs of potentially duplicated TFRs found in the zebrafish genome that were removed from the analysis. [file 1471-2164-8-470-S5.pdf]

Supplemental Table S5: Table showing the presence of TFRs in *X. tropicalis* orthologous to the 58 zebrafish TFRs ≥10 kb found in all mammals.

Fish TFRs		Frog TFRs		
**ID**	**Length (bp)**	**Coordinates including assembly gaps[a]**	**Length (bp)[b]**	**Length with gaps[c]**
dr17.150	35667	scaffold_108:2073443-2091724	7984	18282
dr7.228	14153	scaffold_112:1968634-1990976	22343	22343
dr16.195	16230	scaffold_115:205278-226635	11577	21358
dr5.239	2004	scaffold_122:638743-648547	< 5 kb	9805
dr13.34	7674	scaffold_1423:70788-81103	6092	10316
dr7.190	16312	scaffold_146:2180234-2198209	17976	17976
dr15.2	5896	scaffold_16:3514449-3524152	8616	9704
dr9.4	10292	scaffold_163:559591-592887	24071	33297
dr9.6	26277	scaffold_163:612144-673331	30517	61188
dr13.76	5088	scaffold_173:671733-680453	8721	8721
dr22.74	16342	scaffold_19:4454020-4466388	11037	12369
dr18.135	45099	scaffold_203:964377-1002798	32407	38422
dr19.64	73780	scaffold_213:221861-250722	25919	28862
dr10.2	20198	scaffold_217:655220-699794	< 5 kb	44575
dr7.313	13540	scaffold_22:3991688-4010867	19180	19180
dr25.25	12767	scaffold_22:3991688-4010867	19180	19180
dr23.144	83755	scaffold_226:358499-431638	35169	73140
dr6.14	10123	scaffold_236:617808-635361	17554	17554
dr22.185	17231	scaffold_245:1113196-1143225	30030	30030
dr13.24	27655	scaffold_25:1478824-1507686	7369	28863
dr9.120	24815	scaffold_261:927179-944785	17607	17607
dr17.136	10057	scaffold_263:955582-965590	10009	10009
dr2.66	12210	scaffold_28:101352-112398	8271	11047
dr2.101	13894	scaffold_28:3853156-3868153	< 5 kb	14998
dr7.240	8354	scaffold_28:3853156-3868153	< 5 kb	14998
dr1.27	11995	scaffold_28:3853156-3868153	< 5 kb	14998
dr24.15	25800	scaffold_284:991837-1030987	27929	39151
dr9.136	1745	none	< 5 kb	< 5 kb
dr25.105	44221	scaffold_324:883311-893222	9912	9912
dr3.77	30851	scaffold_334:502923-519002	14346	16080

dr12.132	22969	scaffold_334:540478-563333	22856	22856
dr3.78	15236	scaffold_334:540478-563333	22856	22856
dr12.122	25000	scaffold_334:540478-563333	22856	22856
dr9.10	27729	scaffold_356:712325-724553	12229	12229
dr17.215	80259	scaffold_37:1845502-1865183	19682	19682
dr8.176	5105	scaffold_378:726236-748722	22487	22487
dr15.56	9617	scaffold_388:556851-572918	13734	16068
dr5.206	114930	scaffold_43:1714510-1741794	9863	27285
dr17.234	11616	scaffold_432:752949-766163	10965	13215
dr11.8	5947	scaffold_44:2611867-2640477	15685	28611
dr7.185	38285	scaffold_458:656725-678061	21337	21337
dr2.57	9803	scaffold_47:2624130-2658286	34157	34157
dr24.53	21575	scaffold_47:2624130-2658286	34157	34157
dr24.56	4259	scaffold_47:3002973-3034856	24214	31884
dr17.72	22336	scaffold_473:716782-735931	19150	19150
dr13.114	44922	scaffold_495:119277-132455	13179	13179
dr24.89	30330	scaffold_506:153079-168818	9370	15740
dr24.90	44117	scaffold_506:168930-202537	33608	33608
dr16.87	858	scaffold_51:3140589-3160695	14448	20107
dr18.84	28234	scaffold_53:1016757-1060296	43540	43540
dr19.53	16827	scaffold_56:1370922-1496301	95263	125380
dr16.54	20096	scaffold_56:1370922-1496301	95263	125380
dr19.54	48665	scaffold_56:1370922-1496301	95263	125380
dr16.55	3743	scaffold_56:1370922-1496301	95263	125380
dr5.225	28874	scaffold_58:510914-539730	16249	28817
dr24.76	35350	scaffold_64:1386427-1405117	18691	18691
dr15.25	86655	scaffold_679:149016-165459	12911	16444
dr6.29	13203	scaffold_722:275543-294156	17462	18614
dr9.16	16474	scaffold_722:275543-294156	17462	18614
dr13.12	19193	scaffold_8:4886080-4898488	12409	12409
dr13.11	11552	scaffold_8:4985782-5007431	17018	21650
dr7.55	231	none	< 5 kb	< 5 kb
dr7.272	22991	scaffold_94:1719456-1746642	27187	27187
dr20.101	4964	scaffold_95:2473934-2491458	17525	17525

[a]*X. tropicalis* August 2005 (xenTro2) assembly.

[b]Length of TFR using strict definition where gaps delimit TFRs.

[c]Length of TFRs allowing TFRs to span assembly gaps.

Supplemental Table S6: Pairs of potentially duplicated TFRs found in the zebrafish genome that were removed from the analysis.

TFRs ≥10 kb		Removed duplicate TFRs ≥10 kb	
**ID**	**coordinates**	**ID**	**coordinates**
dr10.133	chr10:29080606-29094280	dr10.132	chr10:29009654-29023286
dr10.165	chr10:43147771-43159583	dr10.166	chr10:44049383-44061114
dr1.112	chr1:30161224-30173470	dr1.103	chr1:29117041-29129285
dr1.112	chr1:30161224-30173470	dr1.105	chr1:29648812-29660892
dr1.112	chr1:30161224-30173470	dr1.113	chr1:30552981-30565225
dr11.88	chr11:26056014-26070150	dr11.91	chr11:26219840-26233975
dr1.190	chr1:48568442-48579472	dr1.194	chr1:48959831-48970861
dr12.135	chr12:34724265-34734369	dr12.124	chr12:34272138-34282229
dr12.133	chr12:34698318-34708359	dr12.125	chr12:34298397-34308397
dr1.199	chr1:50005751-50024837	dr1.214	chr1:51184769-51203854
dr12.187	chr12:51266487-51282428	dr12.189	chr12:51366827-51382682
dr12.187	chr12:51266487-51282428	dr12.191	chr12:51732097-51747956
dr1.222	chr1:51761063-51771277	dr1.219	chr1:51611398-51621602
dr12.41	chr12:18079547-18089955	dr12.51	chr12:20732008-20742218
dr1.27	chr1:8475498-8492906	dr1.26	chr1:8430587-8440853
dr13.34	chr13:12458798-12478075	dr13.37	chr13:12629224-12645187
dr13.34	chr13:12458798-12478075	dr13.38	chr13:12761458-12778334
dr12.4	chr12:1475362-1490577	dr14.176	chr14:52697949-52708422
dr14.214	chr14:65641118-65652778	dr14.221	chr14:65773073-65784733
dr22.177	chr22:40620831-40632048	dr14.300	chr14:81972065-81983033
dr15.102	chr15:30715944-30729219	dr15.103	chr15:30869548-30882820
dr15.147	chr15:36438661-36449725	dr15.150	chr15:36836215-36847279
dr16.182	chr16:54492933-54506467	dr16.203	chr16:65064933-65078278
dr17.116	chr17:25494202-25511816	dr17.113	chr17:25342727-25354835
dr17.21	chr17:2230392-2240650	dr17.13	chr17:1849361-1859584
dr17.136	chr17:30179371-30189406	dr17.142	chr17:30681341-30691363
dr17.165	chr17:37417554-37429293	dr17.190	chr17:40483393-40493410
dr17.161	chr17:37277456-37290023	dr17.196	chr17:40826710-40839215
dr17.14	chr17:1865969-1877967	dr17.20	chr17:2212184-2223973
dr17.234	chr17:44421459-44436173	dr17.228	chr17:43969817-43984513
dr17.235	chr17:44436373-44447608	dr17.229	chr17:43984713-43995947
dr17.234	chr17:44421459-44436173	dr17.231	chr17:44195870-44210566
dr17.235	chr17:44436373-44447608	dr17.232	chr17:44210766-44222000
dr17.245	chr17:46811211-46821685	dr17.246	chr17:46907037-46917511
dr15.69	chr15:25034168-25061004	dr18.159	chr18:52933930-52944719
dr20.170	chr20:44874803-44889102	dr20.109	chr20:32657407-32671693

dr17.150	chr17:35079330-35097548	dr20.196	chr20:60599504-60617722
dr7.43	chr7:20262013-20273532	dr21.167	chr21:37939087-37950166
dr21.173	chr21:41352751-41364188	dr21.172	chr21:41000770-41012206
dr21.176	chr21:41980007-41990299	dr21.177	chr21:42230872-42241164
dr21.47	chr21:15596421-15608043	dr21.53	chr21:15994379-16005978
dr2.189	chr2:41763022-41776615	dr2.176	chr2:39742140-39754660
dr9.6	chr9:1573105-1589323	dr2.20	chr2:12064317-12076545
dr22.177	chr22:40620831-40632048	dr22.105	chr22:24348954-24359172
dr22.161	chr22:37757069-37767142	dr22.152	chr22:37344623-37354687
dr22.177	chr22:40620831-40632048	dr22.62	chr22:17834072-17845245
dr25.78	chr25:26658825-26677387	dr22.79	chr22:19932835-19949135
dr23.173	chr23:49909910-49923137	dr23.170	chr23:49262178-49275167
dr24.76	chr24:26257645-26274018	dr24.82	chr24:27196677-27212984
dr25.117	chr25:37276538-37290101	dr25.119	chr25:37487996-37499171
dr3.117	chr3:29097510-29109075	dr3.111	chr3:28224082-28235624
dr3.117	chr3:29097510-29109075	dr3.122	chr3:29413297-29424862
dr20.110	chr20:33133414-33143760	dr3.285	chr3:70480409-70490663
dr3.297	chr3:75949353-75959491	dr3.290	chr3:74629963-74640018
dr25.57	chr25:19930471-19941503	dr4.354	chr4:36553289-36564316
dr13.90	chr13:27193638-27204587	dr4.80	chr4:9402985-9413930
dr5.236	chr5:61443048-61458187	dr5.240	chr5:62733501-62748610
dr22.177	chr22:40620831-40632048	dr5.241	chr5:63394176-63405257
dr5.239	chr5:62283101-62293242	dr5.243	chr5:67245934-67256003
dr6.14	chr6:8822072-8832216	dr6.15	chr6:8961326-8971470
dr6.213	chr6:59099467-59123108	dr6.184	chr6:51783310-51806852
dr6.248	chr6:68355938-68366329	dr6.190	chr6:53248378-53258719
dr6.213	chr6:59099467-59123108	dr6.210	chr6:58811420-58824263
dr6.248	chr6:68355938-68366329	dr6.238	chr6:65967683-65977953
dr7.344	chr7:73748361-73760445	dr7.345	chr7:73828256-73840340
dr7.84	chr7:27185576-27200976	dr7.70	chr7:22940344-22953494
dr7.84	chr7:27185576-27200976	dr7.85	chr7:27208304-27221929
dr7.98	chr7:29974741-29988432	dr7.97	chr7:29756021-29769690
dr8.75	chr8:24717548-24730163	dr8.192	chr8:63571226-63583776
dr8.24	chr8:14806114-14816168	dr8.25	chr8:15328465-15338519
dr9.136	chr9:29903754-29918951	dr9.133	chr9:29732621-29747746
dr9.181	chr9:40341055-40355848	dr9.182	chr9:40492386-40507179
dr9.78	chr9:21302332-21323748	dr9.79	chr9:21390761-21412177
